# Supplementary material for: Global hypo-methylation in a proportion of glioblastoma enriched for an astrocytic signature is associated with increased invasion and altered immune landscape
Source: eLife. 2022 Nov 22;11:e77335. doi: 10.7554/eLife.77335 (PMC9681209; doi:10.7554/eLife.77335)
Supplement: Figure 2—source data 1. [file elife-77335-fig2-data1.zip › Figure_2_source_data_1/Figure_2I_J/homerResults/motif19.similar.html]

motif19

## Information for motif19

C
T
A
G
A
T
C
G
C
T
G
A
T
A
G
C
C
T
G
A
T
A
G
C
G
T
C
A
A
T
G
C
T
A
G
C
G
C
A
T
C
A
T
G
A
T
C
G
T
A
C
G
A
G
T
C
T
A
C
G
A
C
G
T
T
C
A
G
A
C
G
T
T
A
G
C
A
G
T
C
  
Reverse Opposite:  

C
T
A
G
A
T
C
G
T
G
C
A
A
G
T
C
T
G
C
A
A
T
G
C
T
C
A
G
A
T
G
C
T
A
G
C
G
T
A
C
C
G
T
A
A
T
C
G
T
A
C
G
A
C
G
T
A
T
C
G
G
A
C
T
A
T
C
G
G
A
C
T
T
A
G
C
G
A
T
C
  

|  |  |
| --- | --- |
| p-value: | 1e-37 |
| log p-value: | -8.741e+01 |
| Information Content per bp: | 1.544 |
| Number of Target Sequences with motif | 44.0 |
| Percentage of Target Sequences with motif | 6.06% |
| Number of Background Sequences with motif | 0.4 |
| Percentage of Background Sequences with motif | 0.15% |
| Average Position of motif in Targets | 97.1 +/- 48.2bp |
| Average Position of motif in Background | 44.0 +/- 0.0bp |
| Strand Bias (log2 ratio + to - strand density) | 0.4 |
| Multiplicity (# of sites on avg that occur together) | 1.28 |
| Motif File: | file (matrix) reverse opposite |

### Similar de novo motifs found

|  |  |  |  |  |  |  |  |
| --- | --- | --- | --- | --- | --- | --- | --- |
| Rank | Match Score | Redundant Motif | P-value | log P-value | % of Targets | % of Background | Motif file |
| 1 | 0.833 | T C A G A T C G T C G A A G T C T C G A A T G C T C G A A T G C T A G C A G C T C T A G T C A G T A C G A G T C T A C G A G C T T C A G C G A T A T G C G A T C A T C G T A G C G A T C G A C T T A G C | 1e-37 | -87.406215 | 6.06% | 0.61% | motif file (matrix) |
| 2 | 0.699 | C T G A A T G C T G C A A G T C A T G C A C G T A T C G C A G T A T C G A G T C | 1e-21 | -49.285849 | 4.13% | 0.56% | motif file (matrix) |
| 3 | 0.678 | T A C G A T C G T G C A T A G C T C G A T A G C C T G A T A G C T A G C G A C T C A T G T C A G T A C G G T A C T A G C G A C T T A G C A G C T T A G C A G T C A T C G T A G C T G A C G A C T A T G C G A T C G A C T A G T C T G A C A G T C | 1e-11 | -25.580014 | 3.72% | 0.99% | motif file (matrix) |
| 4 | 0.725 | T G A C A G T C A T G C G A T C T C A G A C T G T A C G T A C G A T C G T G C A A G T C T G C A A G T C T C G A T G A C A G T C G A C T A T C G C T A G A T C G A T G C A T C G G A C T T C A G A C G T A T G C G A T C T A C G A G T C A G T C G C A T A T G C T A G C A G C T G T A C | 1e-9 | -21.108818 | 4.13% | 1.21% | motif file (matrix) |
